# Recurrent child mortality risks and parity transition in Nigeria

**DOI:** 10.1186/s12978-019-0733-6

**Published:** 2019-06-07

**Authors:** Abiodun Idowu Adanikin, Sabu S. Padmadas, Nuala McGrath

**Affiliations:** 1 Department of Social Statistics and Demography and Centre for Global Health, Population, Poverty and Policy, University of Southampton, Southampton, UK; 2 Department of Reproductive Health and Research, World Health Organization, Geneva, Switzerland; 3 Primary Care and Population Studies Academic Unit, Faculty of Medicine, University of Southampton, Southampton, UK

**Keywords:** Fertility, Child mortality, Nigeria, Sub-Saharan Africa

## Abstract

**Background:**

Fertility rates remain persistently high in Nigeria, with little difference across socioeconomic groups. While the desire for large family size is culturally rooted, there is little understanding of how repeated child mortality experiences influence fertility behaviour and parity transition in Nigeria.

**Methods:**

Using birth history data from the 2013 Nigeria Demographic and Health Survey (NDHS), we applied life table techniques and proportional-hazard regression model to explore the effect of child survival experience on parity transitions. We hypothesize that a woman with one or more child death experience is at elevated risk of progressing towards higher parities.

**Results:**

Our findings show that child mortality is concentrated among mothers living in deprived conditions especially in rural areas of the northern part of Nigeria and among those with little or no education and, among those belonging to Hausa/Fulani ethnicity and Islam religion. Mothers with repeated experience of child deaths were significantly at a higher rate of progressing to higher parities than their counterparts (HR: 1.45; 95% CI: 1.31–1.61), when adjusted for relevant biological and socio-demographic characteristics.

**Conclusion:**

Recurrent experience of child deaths exacerbates the risks to higher parity transition. Interventions aimed at reducing fertility in Nigeria should target promoting child survival and family planning concurrently.

## Plain English summary

In Nigeria, having many children is seen as a source of pride in the community. When the possibility that a child dies is high, a woman may either decide to have more children than she needs as an insurance or wait until the child dies and then have another one to replace him/her. In this study, we examine how the experience of child death influences the reproductive behaviour of women in Nigeria, particularly the decision to replace a dead child.

We analysed the birth record of women using the 2013 Nigeria Demographic and Health Survey. Our results revealed that child death was commoner among women who have no education, who are Hausa/Fulani, who live in rural areas or northern Nigeria, and among those who are Muslims. Mothers who had experienced repeated child loss had a higher chance of having another birth to replace the dead ones, even after adjusting for other attributes.

This study reiterates the need to prioritize health interventions that ensure child survival alongside the promotion of family planning use in Nigeria if fertility rate will reduce.

## Background

Nigeria remains one of the countries with high total fertility rates (TFR). This found socio-cultural explanations in the practice of polygyny in most regions in the country, and having a large family which is culturally symbolic as a proof of social standing [1]. There is also widespread child fosterage by extended families and provision of affordable childcare including community kindergartens [2, 3]. The demand of work force for subsistence agriculture is yet another factor associated with high fertility in Nigeria [1, 3, 4]. These attributes are deeply entrenched within the existing cultural norm and have been resistant to change. However, a demographic explanation relates to the frequency and clustering of child mortality in Nigerian families. The strong desire for large families could motivate couples to continue reproduction and replace dead children until the desired family size is attained.

When the risk of child mortality is high, there are two response mechanisms with behavioural and cultural implications [5, 6]. The first response is replacement behaviour, which is a deliberate and conscious effort of couples to replace a dead child. The second response is hoarding, which is having more children than desired as an insurance against future child mortality [5, 7, 8]. Couples who want to replace or hoard children are less likely to use contraception or may be more likely to cease its usage in order to get pregnant [9, 10]. Another mechanism described by which child mortality leads to high fertility is the short-term physiological changes following the cessation of breastfeeding, return of ovulation and subsequent increased vulnerability to getting pregnant [5]. In certain situations, the physiological and replacement response could be concomitant [11].

Mostly, researches on child mortality and fertility behaviour in Nigeria have focused on risk factors and causes of deaths among Nigerian children [12], regional trends and variations in child mortality [13–15], survival probability and predictors of a woman experiencing child death [16], and the impact of desired fertility on fertility behaviour [17]. There is however dearth of research evidence on the impact of child mortality experience on reproductive behaviour in Nigeria. This study aims to investigate the risk of transiting towards higher fertility when a woman experiences one or more child death. The understanding gained from this study can inform the design of appropriate child survival programs and family planning programs that synergistically work at curtailing high fertility in Nigeria.

### Framework outlining the relationship between child death and fertility

**Fig. 1:**
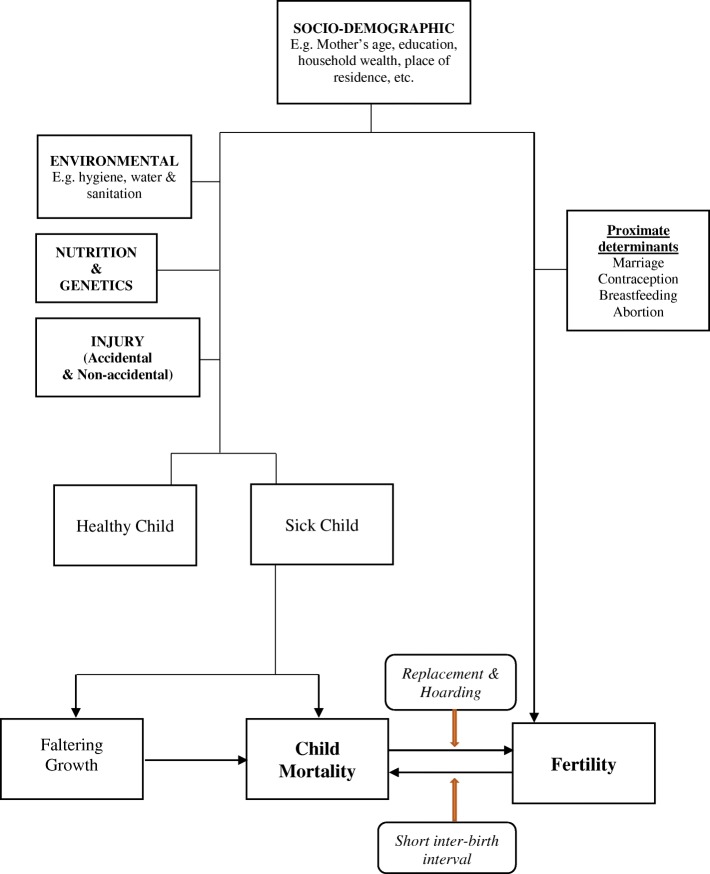
*Framework showing relationship between child mortality and fertility*

Interpreting the relationship between child mortality and fertility can be inherently complex as they seem causally linked in both directions (Fig. 1). Similar socio-economic and demographic factors affect fertility and child mortality [18]. But in addition to these, proximate determinants such as marriage, age at first cohabitation, postpartum infecundability (lactational amenorrhea), contraceptive use and abortion are crucial in determining a woman’s total fertility [19, 20]. In contrast, the health status of a child is determined not only by household socio-economic conditions and environmental factors but also by nutrition, genetics and exposure to injuries [21]; these can make an unhealthy child to falter in growth and/or die during early years of life.

To establish a causal link, the endogeneity of both births and deaths makes interpretation challenging, especially as it relates to hoarding effect. High fertility could increase child mortality through shorter inter-birth interval [22]. On the other hand, child mortality can lead to cessation of breastfeeding, resumption of ovulation and, increase the chance of conception and childbearing [18, 23, 24]. In analysing replacement effect, time-to-event analysis can be adopted [9]. Given the problems of simultaneity, we need to exercise caution while drawing inference on the contribution of child mortality to total fertility. It is likely that child deaths may be higher for a woman simply because she had more children, though her child mortality rate was the same as others [25]. That notwithstanding, evidence of child replacement in a society is alone sufficient to generate a positive relationship between child mortality and total fertility [26].

## Methods

The analysis is based on the individual birth histories drawn from the most recent (2013) Nigeria Demographic and Health Survey (NDHS). The 2013 NDHS was the fifth survey implemented by the Nigeria Population Commission (NPC), after the consecutive rounds carried out in 1990, 1999, 2003 and 2008. The overarching goal of the 2013 NDHS was to provide quality data for monitoring the population and health situation in Nigeria especially maternal and child health and family planning services [27].

Three questionnaires were implemented in the 2013 NDHS: household, women and men. The women’s questionnaire was administered to all women aged 15–49 years in a nationally representative sample of 40,680 households. The households were selected using a three-stage stratified design consisting of 904 clusters. All eligible women aged 15–49 years who were permanent or usual residents *(de jure)* or visitors who slept in the selected households overnight *(*de facto*)* were interviewed. The questionnaire obtained information on their background characteristics, birth history, family planning practices, fertility preferences, and so on. The birth history data provide detailed information of each birth including birth order, month and year of birth, sex of the child, survivorship status, and age at death.

The primary outcome of interest in this study was *parity transition*, defined as a successful transition from one parity to another, from 3 to 4, 4 to 5, 5 to 6 and 6 to 7 children. Our analysis focuses on high parity behaviours and hence we consider parity 3 and higher for the analysis, also reflecting on levels of current fertility in Nigeria. For each of the specific parity considered, a successful transition to the next parity was classified as an ‘event’. In situations where transition to the next parity (event) had not occurred at the time of the survey, the event in question was right ‘censored’. In order to understand a woman’s progression to subsequent parity, the survival status of the immediate preceding child (IPC), and older children, if any, was considered. The index birth is the birth of IPC. The explanatory variable was therefore coded into five sub-categories: *all children alive*; *immediate preceding child (IPC) alone died*; *IPC and a previous child [ren] died*; *IPC alive but a previous child [ren] died since index birth*; and, *IPC alive but a previous child [ren] died before index birth*.

**Fig. 2:**
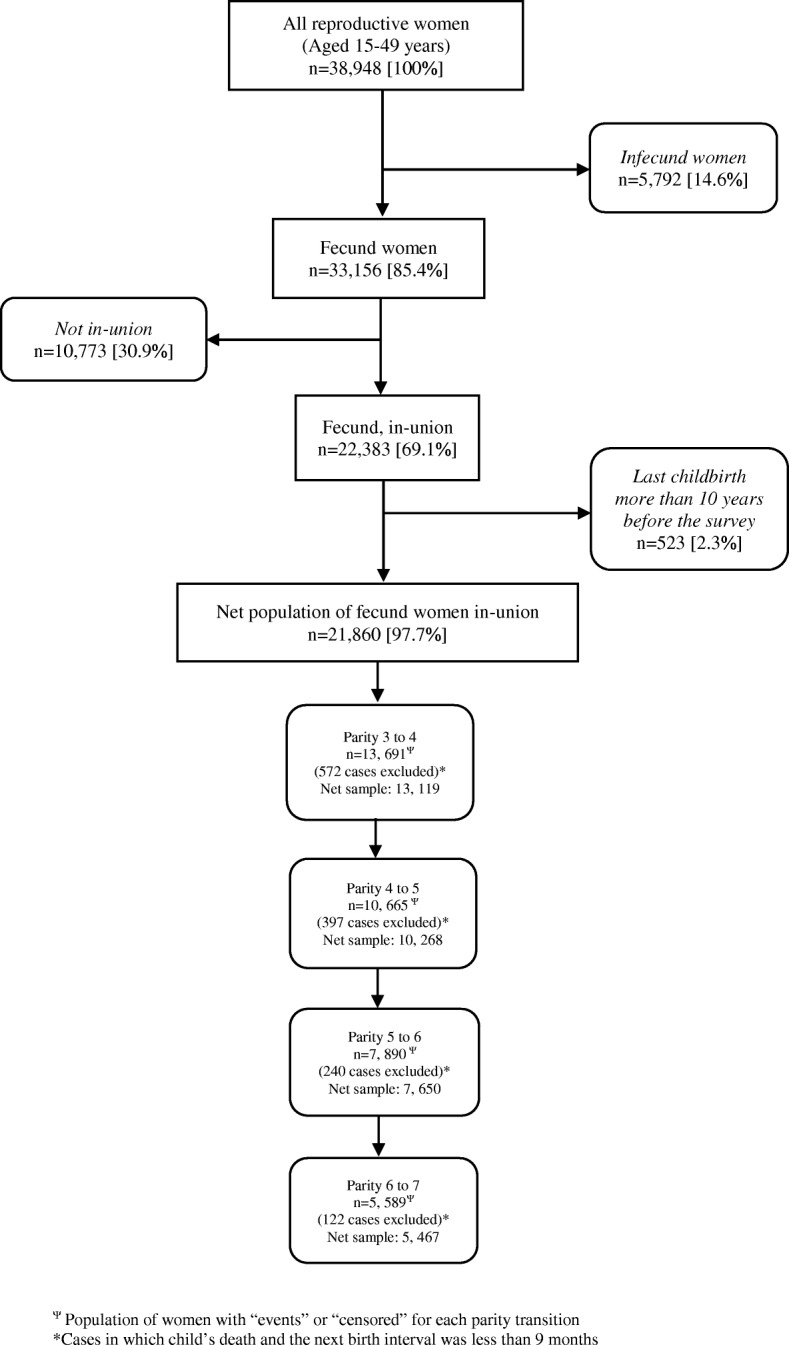
*Case selection from the 2013 NDHS individual women’s data (% weighted)*

Our study population consists of fecund women who are currently in a marital union or cohabiting. The analysis was restricted to a period of 10 years prior to the date of interview (survey) to capture recent trends in parity progression, and to leave enough time for the women to make birth transitions [19]. The 10-year period also ensures reliability of birth history data and reduces potential bias in misreporting the dates of birth of older children. Out of the 22,383 reproductive women reported to be currently in-union, 523 women who had their last birth more than 10 years before the survey were excluded. A flow chart showing the selection of sample for the analysis is shown in Fig. 2.

To consider the impact of child (ren)‘s death on the subsequent birth, for each of the parities analysed, births that occurred less than 9 months after a child’s death were excluded. The assumption of minimum 9 months takes into account of the factors that influence the length of inter-birth intervals including post-partum infecundability and waiting time to conception in the absence of contraceptive use. However, since the decision to have another child could be triggered by protracted illness of a child, even before the child’s demise, we conducted sensitivity analysis without excluding cases in which the duration between child’s death and next birth was less than 9 months.

Pearson’s chi-squared test (χ2) was used to examine the association between child mortality and women’s characteristics. Then, we applied a Cox proportional hazard regression to predict the hazard of parity transition, given a specific group of independent variables. The selection of control variables for the analysis was guided by the literature [18, 24]. We adjusted for women’s socio-demographic attributes (including her age, place and region of residence, ethnicity, religion, educational status and wealth status). Though we considered proximate fertility determinants such as postpartum infecundability and marital status for determining the study sample inclusion criteria, we further controlled for age at first cohabitation and the number and type of conjugal unions [19]. Since the desire for large family size is widely prevalent across Africa including Nigeria [2, 4], we controlled for women’s and their partner’s fertility preferences. In addition, we adjusted for the duration of inter-birth intervals (IBIs) as proxy factor for potential biological effects.

The same variables were retained in the final multivariate model for each parity transition in an effort to aid cross comparison. We defined ‘*survival time*’ as the interval (in months) between the current (index) birth and the succeeding birth for those who made the parity transition, and the time difference (in months) between the last birth and the date of interview for those who did not transit to next parity. We tested that proportionality assumption was satisfied by the explanatory variables using partial residuals and log minus log plot. The analysis was carried out in SPSS v.22.

## Results

Table 1 summarises the women’s child mortality experience by parity transition. A total of 13,119 women experienced a transition from parity 3 to 4, of which 10,268 made further transition from parity 4 to 5, and so on. More women with history of child mortality constituted the group making higher parity transition. For instance, the prevalence of child mortality was 34% among those who made a transition from parity 3 to 4, however, it was 64% among women moving from parity 6 to 7.

**Table 1:** Child mortality experience by parity transition in Nigeria (2013 DHS)

Variable	Parity 3 to 4	Parity 4 to 5	Parity 5 to 6	Parity 6 to 7
Base population	*13,119 (100)*	*10,268 (100)*	*7650 (100)*	*5467 (100)*
Child mortality experience				
No	8560 (65.7)	5620 (55.3)	3456 (45.5)	1969 (36.5)
Yes	4559 (34.3)	4648 (44.7)	4194 (54.5)	3498 (63.5)
Conditional on child mortality				
Immediate preceding child (IPC) alone died	597 (4.4)	394 (3.8)	215 (2.7)	125 (2.2)
IPC + a previous child [ren] died	994 (7.6)	985 (9.6)	845 (10.8)	676 (12.2)
IPC is alive, but a previous child [ren] died *since index birth*	236 (1.8)	225 (2.3)	195 (2.5)	157 (2.9)
IPC is alive, but a previous child [ren] died *before index birth*	2732 (20.5)	3044 (29.0)	2939 (38.6)	2540 (46.1)

Data presented as n (%)

The percentage is adjusted for sample weight

The women’s attributes by child mortality experience are presented in Table 2. Child deaths are concentrated more among older women, those without any formal education, those living in rural areas and/or the northern region of Nigeria, those who are of Hausa/Fulani ethnicity, practicing Islam, from the poorest wealth strata, who married early and with history of short inter-birth intervals.

**Table 2:** Child mortality experience by women’s attributes (*n* = 13,119) – Nigeria 2013 DHS

Variables	No child mortality, n (%)	Previous child mortality, n (%)	χ2 (*P* value)
Current age group (years)			149.5 (< 0.001)
15–24	647 (62.0)	417 (38.0)	
25–29	1951 (60.6)	1299 (39.4)	
30–34	1978 (58.8)	1398 (41.2)	
35–39	1566 (53.4)	1416 (46.6)	
40–44	841 (50.0)	888 (50.0)	
45–49	301 (42.6)	417 (57.4)	
Educational Status			566.4 (< 0.001)
No education	2922 (47.4)	3444 (52.6)	
Primary	1768 (58.1)	1288 (41.9)	
Secondary or higher	2028 (71.6)	935 (28.4)	
Place of residence			312.2 (< 0.001)
Urban	2884 (67.2)	1458 (32.8)	
Rural	4400 (50.4)	4377 (49.6)	
Region of residence			274.7 (< 0.001)
South	2864 (66.7)	1494 (33.3)	
North	4420 (51.5)	4341 (48.5)	
Ethnicity			384.2 (< 0.001)
Other tribes	4974 (64.1)	3007 (35.9)	
Hausa/Fulani	2306 (46.0)	2828 (54.0)	
Religion			219.4 (< 0.001)
Christianity & Others	3433 (64.5)	2002 (35.5)	
Islam	3851 (51.3)	3833 (48.7)	
Wealth status			724.6 (< 0.001)
Poorest	1242 (42.7)	1765 (57.3)	
Poorer	1380 (47.3)	1603 (52.7)	
Middle	1551 (59.5)	1046 (40.5)	
Richer	1596 (65.6)	883 (34.4)	
Richest	1515 (74.5)	538 (25.5)	
Age at first cohabitation (years)			452.5 (< 0.001)
≤ 14	1662 (44.6)	2130 (55.4)	
15–18	2806 (56.4)	2322 (43.6)	
19–24	2152 (65.8)	1133 (34.2)	
≥ 25	664 (73.4)	250 (26.6)	
Number of union(s)			140.8 (< 0.001)
1	6535 (58.3)	4820 (41.7)	
≥ 2	749 (42.1)	1015 (57.9)	
*Type of union*			202.5 (< 0.001)
Monogyny	4980 (61.2)	3285 (38.8)	
Polygyny	2304 (47.9)	2550 (52.1)	
Perceived ideal fertility			346.4 (< 0.001)
≤ 3	309 (65.1)	158 (34.9)	
4–5	2171 (69.0)	980 (31.0)	
≥ 6	4202 (51.3)	4153 (48.7)	
Non-numeric response	602 (55.8)	544 (44.2)	
Partner’s fertility preference			126.3 (< 0.001)
Partner wants same/fewer children	4208 (60.1)	2889 (39.9)	
Partner wants more children	2767 (50.7)	2775 (49.3)	
Unsure	309 (67.5)	171 (32.5)	
Had short inter-birth intervals (IBIs)			863.9 (< 0.001)
No	3316 (73.5)	1223 (26.5)	
Yes	3968 (47.2)	4612 (52.8)	

The percentage is adjusted for sample weight

Table 3 reports the adjusted Cox regression models illustrating the relationship between the hazard of parity transition and selected women’s characteristics. After adjusting for biological factors, marital attributes and socio-demographic variables, child death remains the strongest factor influencing parity transition. Specifically, women who had experienced multiple child losses, IPC and older child, were approximately 1.4 times likely to progress to next parity when compared to those without any child loss and this was consistent across parities. Although a ‘one-off’ death involving the IPC had no consistent pattern of influence on the rate of parity transitions, relative risk (RR) of parity transition is higher than 1 when a multiple child loss involved an IPC, compared to when IPC survived but a previous child died since index birth. In the scenario that IPC survived but a previous child (ren) died since index birth, the progression to having another birth was delayed. Although the experience of child bereavement predating a woman’s last childbirth had potential to influence parity transition, it was not in every occasion. Moreover, whilst women with history of short IBIs (< 2 years) were likely to have higher parity transition, short IBI was inconsistent as a predictive factor and the effect size smaller compared to multiple child loss.

**Table 3:** Adjusted hazard ratios from cox regression models showing the relationship between higher parity transitions and child mortality experience

Variables	Hazard ratios (95% CI)			
	Parity 3 to 4	Parity 4 to 5	Parity 5 to 6	Parity 6 to 7
Child Mortality Experience				
All children alive (Ref)				
Immediate preceding child (IPC) alone died	1.04 (0.95–1.14)	**1.25 (1.11–1.40)**	1.01 (0.86–1.19)	1.25 (1.00–1.56)
IPC + a previous child [ren] died	**1.36 (1.27–1.47)**	**1.39 (1.29–1.51)**	**1.40 (1.28–1.52)**	**1.45 (1.31–1.61)**
IPC is alive, but a previous child [ren] died since index birth	**0.78 (0.69–0.89)**	**0.85 (0.74–0.98)**	**0.83 (0.72–0.97)**	**0.75 (0.63–0.88)**
IPC is alive, but a previous child [ren] died before index birth	**1.09 (1.03–1.14)**	1.05 (1.00–1.11)	**1.10 (1.01–1.11)**	1.09 (1.00–1.18)
Current age group (years)				
15–24	**1.34 (1.18–1.52)**	1.25 (0.97–1.60)	**3.70 (1.92–7.15)**	1.25 (0.17–8.95)
25–29	**1.07 (1.01–1.14)**	1.05 (0.97–1.13)	1.04 (0.93–1.17)	1.22 (1.00–1.47)
30–34 *(Ref)*				
35–39	**0.90 (0.85–0.95)**	**0.88 (0.82–0.93)**	**0.89 (0.82–0.95)**	0.92 (0.83–1.01)
40–44	**0.83 (0.77–0.88)**	**0.77 (0.72–0.83)**	**0.80 (0.74–0.87)**	**0.80 (0.72–0.89)**
45–49	**0.73 (0.67–0.80)**	**0.69 (0.63–0.76)**	**0.69 (0.62–0.77)**	**0.73 (0.64–0.83)**
Educational Status				
No education (Ref)				
Primary	0.99 (0.93–1.05)	0.98 (0.91–1.04)	0.95 (0.87–1.02)	0.99 (0.90–1.10)
Secondary or higher	0.93 (0.87–1.00)	0.99 (0.91–1.08)	0.94 (0.83–1.04)	0.97 (0.85–1.11)
Place of residence				
Urban (Ref)				
Rural	1.00 (0.94–1.05)	0.96 (0.90–1.02)	1.01 (0.94–1.09)	0.96 (0.87–1.05)
Region of residence				
South (Ref)				
North	0.98 (0.93–1.04)	1.02 (0.95–1.09)	0.94 (0.86–1.03)	**0.86 (0.77–0.97)**
Ethnicity				
Other tribes (Ref)				
Hausa/Fulani	**1.12 (1.05–1.19)**	1.05 (0.98–1.12)	1.08 (1.00–1.17)	1.05 (0.95–1.15)
Religion				
Christianity & Others (Ref)				
Islam	1.00 (0.94–1.06)	1.00 (0.93–1.08)	1.04 (0.95–1.14)	1.28 (1.14–1.44)
Wealth status				
Poorest (Ref)				
Poorer	0.98 (0.93–1.04)	0.94 (0.88–1.01)	1.05 (0.98–1.14)	0.97 (0.89–1.06)
Middle	**0.92 (0.86–0.98)**	**0.88 (0.82–0.95)**	0.93 (0.85–1.02)	0.96 (0.86–1.07)
Richer	**0.90 (0.83–0.97)**	**0.86 (0.78–0.94)**	0.90 (0.81–1.00)	**0.81 (0.71–0.93)**
Richest	**0.83 (0.75–0.92)**	**0.80 (0.72–0.90)**	**0.83 (0.72–0.96)**	**0.80 (0.66–0.96)**
Age at first cohabitation (years)				
≤ 14	**0.80 (0.73–0.89)**	**0.77 (0.68–0.88)**	**0.76 (0.64–0.90)**	0.90 (0.72–1.11)
15–18	**0.84 (0.76–0.92)**	**0.82 (0.72–0.93)**	**0.82 (0.70–0.97)**	0.94 (0.76–1.16)
19–24	0.92 (0.84–1.01)	**0.85 (0.75–0.97)**	**0.77 (0.65–0.92)**	1.00 (0.80–1.25)
≥ 25 (Ref)				
Number of union(s)				
1 (Ref)				
≥ 2	**0.91 (0.85–0.96)**	**0.91 (0.85–0.97)**	1.01 (0.93–1.09)	0.93 (0.85–1.01)
Type of union				
Monogyny (Ref)				
Polygyny	0.96 (0.92–1.01)	0.97 (0.93–1.02)	0.99 (0.93–1.05)	**0.92 (0.86–0.99)**
Perceived ideal fertility				
≤ 3 (Ref)				
4–5	0.98 (0.85–1.12)	**0.77 (0.65–0.92)**	1.13 (0.92–1.40)	0.92 (0.71–1.19)
≥ 6	1.13 (0.99–1.29)	0.97 (0.82–1.15)	1.12 (0.92–1.36)	0.96 (0.76–1.20)
Non-numeric response	**1.17 (1.01–1.35)**	1.02 (0.85–1.22)	1.17 (0.95–1.44)	0.97 (0.76–1.24)
Partner’s fertility preference				
Partner wants same/fewer children (Ref)				
Partner wants more children	1.02 (0.98–1.07)	1.04 (0.99–1.09)	1.02 (0.96–1.08)	**0.91 (0.85–0.98)**
Unsure	0.97 (0.87–1.09)	0.93 (0.81–1.07)	1.01 (0.84–1.22)	0.91 (0.72–1.16)
Inter-birth intervals (IBIs)				
All prior IBIs < 2 years	**1.27 (1.19–1.36)**	**1.22 (1.09–1.36)**	1.19 (1.00–1.41)	**1.37 (1.04–1.80)**
Some prior IBIs < 2 years	**1.12 (1.08–1.17)**	**1.10 (1.04–1.15)**	**1.09 (1.01–1.17)**	1.04 (0.94–1.15)
All prior IBIs ≥2 years (Ref)				

The values shown in bold were statistically significant at *p* < 0.05

The hazard associated with higher parities, irrespective of child mortality experience, tend to be lower for women after age 35, and rather consistently for those belonging to the highest wealth strata. The place of residence and religion were not significantly associated with higher parity transition. The sensitivity analysis that included cases in which the interval between child’s death and next birth was less than 9 months yielded similar results (not shown separately).

## Discussion

This paper investigated birth transition among married women of reproductive ages in Nigeria by their child survival experience and socio-demographic attributes. We found that repeat child mortality involving the IPC was the strongest hazard for higher parity transitions, even after adjusting for other factors. The preponderance of child mortality was among socially and economically disadvantaged women – among the poor and rural residents, and certain ethnic and religious groups, particularly the Hausa/Fulani ethnicity and Muslims.

The foregoing analysis result provides evidence to support the hypothesis of replacement behaviour to overcome child mortality and confirms that recurrent child mortality increases transition to higher parities in Nigerian women. It suggests that improvement in child survival could contribute to lowering fertility in Nigeria, and even possibly elsewhere. Indeed, in Ethiopia there was higher chance of conception in months following death of an index child, with the fertility response being strongest after the death of fourth or fifth child [24]. Similarly, death of index child increased the chances of a woman having another birth in Kenya, Lesotho, Malawi, Tanzania and Zimbabwe [18], though it is noteworthy that in our research such influence is only consistent within the context of recurrent child losses.

Our research finding mostly agrees with the observation by Lindstrom and Kiros [24], that the death of non-index child had no influence on parity transition. It is possible more allowance for time to mourn the demise of an older child, and postpartum infecundability determined by breastfeeding of a younger child account for the delay in parity transition. Also, interesting to note is the finding that short IBIs was not consistent in predicting transition to higher fertility. It is possible that a woman can have rapid births to enable her resume career pursuit or her education in time. The reverse is equally plausible - as a compensation for delay in childbearing owing to career or academic endeavour, a woman can have short IBIs without necessarily progressing to high parity.

Studies have reported differences in rural-urban healthcare access across different geographical regions [28], and Nigeria is not an exception [29]. The geographical variation in access to health care is known to affect fertility, contraceptive use and child health outcomes. In addition, the rural-urban difference in child mortality can also be explained from disadvantage in household characteristics, such as lack of electricity, safe water deprivation, lack of basic amenities and community-level infrastructure [30].

Poverty usually deters women from seeking antenatal care, childbirth and postnatal care, and can adversely influence the survival outcomes of children. Our findings demonstrate that household wealth has a negative effect on parity transition. Previous studies have also demonstrated that household wealth status impacts fertility behaviour in Nigeria and that women from wealthier households had fewer children [3, 31]. Additionally, the higher rates of mortality related to early marriage (widely practiced in northern Nigeria) as found in this study is well known [1], and possibly compounded by lack of autonomy to make decisions related to family planning. By reducing early marriage, and enhancing women’s agency to make family planning decisions, child mortality can be lowered.

Our analysis is based on the latest (2013) Nigerian Demographic Health Survey data and the first of its kind within the Nigerian context to systematically and critically examine the topic, disaggregating the contribution of child mortality to birth transition across the reproductive life course. But, the study is not exempt from limitations. There is a possibility that the characteristics of the women (e.g. place of residence, region of residence, educational attainment, etc.) may not have been static over the course of their parity transitions, as we were constrained to use the cross-sectional information as recorded during the survey. However, the adoption of an observation window in the analysis correctly captured recent trends in parity transition thereby strengthening the inferences made. We also acknowledge that since child mortality was retrospectively reported by mothers during the survey, it is susceptible to memory lapse and reporting bias.

## Conclusion

Recurrent child mortality mostly affects socially and economically disadvantaged women in Nigeria and influences higher parity transition. Any interventions aimed at reducing fertility in Nigeria should target socio-economically disadvantaged mothers and those experiencing child loss at the start of their reproductive careers to prevent repeat child loss and a tendency to transit to higher parity.

### Policy implication

The results provide directions for targeted policy and programmatic interventions, especially as it relates to maternal and child health. Long term fertility reduction may be unlikely without strengthening child survival chance, while promoting contraceptive uptake. Apart from providing free child health services across the country which will assist the poorest to access care for an ill child, radical health promotion in the area of child nutrition and hygiene may also be needed. It is possible that when child mortality in Nigeria is brought to barest minimum, compensatory reduction in fertility through effective use of long-term contraceptives will occur.

## Abbreviations

IBI: inter-birth interval
IPC: Immediate preceding child
NDHS: Nigeria demographic and health survey

## References

[1] Izugbara CO, Ezeh AC (2010). Women and high fertility in Islamic northern Nigeria. Stud Fam Plan.

[2] Korotayev A, Zinkina J, Goldstone J, Shulgin S (2016). Explaining current fertility dynamics in tropical Africa from an anthropological perspective: a cross-cultural investigation. Cross-Cult Res.

[3] Mberu BU, Reed HE (2014). Understanding subgroup fertility differentials in Nigeria. Popul Rev.

[4] Caldwell John C., Orubuloye I. O., Caldwell Pat (1992). Fertility Decline in Africa: A New Type of Transition?. Population and Development Review.

[5] Gyimah SO, Fernando R (2002). The effects of infant deaths on the risk of subsequent birth: a comparative analysis of DHS data from Ghana and Kenya. Soc Biol.

[6] Angeles L (2010). Demographic transitions: analyzing the effects of mortality on fertility. J Popul Econ.

[7] Feyisetan BJ, Bankole A (2009). Fertility transition in Nigeria: trends and prospect. Completing the fertility transition.

[8] LeGrand T, Koppenhaver T, Mondain N, Randall S (2003). Reassessing the insurance effect: a qualitative analysis of fertility behavior in Senegal and Zimbabwe. Popul Dev Rev.

[9] Gyimah SO, Rajulton F (2004). Intentional replacement of dead children in sub-Saharan Africa: evidence from Ghana and Kenya. Canadian Studies in Population.

[10] Rahman Mizanur (1998). The Effect of Child Mortality on Fertility Regulation in Rural Bangladesh. Studies in Family Planning.

[11] Tymicki K. The interplay between infant mortality and subsequent reproductive behaviour. Evidence for the replacement effect from historical population of Bejsce parish, 18 th-20 th centuries, Poland. Historical Social Research/Historische Sozialforschung. 2005:240–64.

[12] Kayode GA, Adekanmbi VT, Uthman OA (2012). Childbirth: risk factors and a predictive model for under-five mortality in Nigeria: evidence from Nigeria demographic and health survey. Bmc Pregnancy and Childbirth.

[13] Adedini SA, Odimegwu C, Imasiku EN, Ononokpono DN, Ibisomi L (2015). Regional variations in infant and child mortality in Nigeria: a multilevel analysis. J Biosoc Sci.

[14] Akinyemi JO, Bamgboye EA, Ayeni O (2015). Trends in neonatal mortality in Nigeria and effects of bio-demographic and maternal characteristics. BMC Pediatr.

[15] Adebayo SB, Fahrmeir L (2005). Analysing child mortality in Nigeria with geoadditive discrete-time survival models. Stat Med.

[16] Adebowale AS, Yusuf BO, Fagbamigbe AF (2012). Survival probability and predictors for woman experience childhood death in Nigeria: analysis of north–south differentials. BMC Public Health.

[17] Bankole A (1995). Desired fertility and fertility behaviour among the Yoruba of Nigeria: a study of couple preferences and subsequent fertility. Popul Stud.

[18] Bungu ML: Two investigations into the causal link between child mortality and subsequent fertility using DHS data from Kenya, Lesotho, Malawi, Tanzania and Zimbabwe. University of Cape Town; 2013.

[19] Hinde A. Demographic methods: Routledge; 2014.

[20] Bongaarts John, Frank Odile, Lesthaeghe Ron (1984). The Proximate Determinants of Fertility in Sub-Saharan Africa. Population and Development Review.

[21] Mosley W. Henry, Chen Lincoln C. (1984). An Analytical Framework for the Study of Child Survival in Developing Countries. Population and Development Review.

[22] DaVanzo J, Hale L, Razzaque A, Rahman M (2008). The effects of pregnancy spacing on infant and child mortality in Matlab, Bangladesh: how they vary by the type of pregnancy outcome that began the interval. Popul Stud.

[23] Obonyo B, Otieno F, Muga R: Effect of Infant and Child Mortality on fertility in Kenya. In: Woking Paper vol. 2: National coordinating agency for population and development; 2005.

[24] Lindstrom DP, Kiros G-E (2007). The impact of infant and child death on subsequent fertility in Ethiopia. Popul Res Policy Rev.

[25] Bhat M (1998). Micro and macro effects of child mortality on fertility: the case of India.

[26] Doepke M (2005). Child mortality and fertility decline: does the Barro-Becker model fit the facts?. J Popul Econ.

[27] NPC [Nigeria] and ICF International: Nigeria Demographic and Health Survey 2013. In*.* Abuja, Nigeria and Rockville, Maryland, USA: NPC and ICF International.; 2014.

[28] Say L, Raine R (2007). A systematic review of inequalities in the use of maternal health care in developing countries: examining the scale of the problem and the importance of context. Bull World Health Organ.

[29] Awofeso N (2010). Improving health workforce recruitment and retention in rural and remote regions of Nigeria. Rural Remote Health.

[30] Van De Poel E, O'donnell O, Van Doorslaer E (2009). What explains the rural-urban gap in infant mortality: household or community characteristics?. Demography.

[31] Lamidi EO (2015). State variations in Women’s socioeconomic status and use of modern contraceptives in Nigeria. PLoS One.

